# Comparative genomics of three non-hematophagous leeches (*Whitmania* spp.) with emphasis on antithrombotic biomolecules

**DOI:** 10.3389/fgene.2025.1548006

**Published:** 2025-02-26

**Authors:** Fang Zhao, Zuhao Huang, Lizhou Tang, Wenting Zhang, Zichao Liu, Gonghua Lin

**Affiliations:** ^1^ School of Life Sciences, Key Laboratory of Jiangxi Province for Functional Biology and Pollution Control in Red Soil Regions, Jinggangshan University, Ji’an, China; ^2^ College of Life Sciences, Jiangxi Normal University, Nanchang, China; ^3^ Engineering Research Center for Exploitation and Utilization of Leech Resources in Universities of Yunnan Province, School of Agronomy and Life Sciences, Kunming University, Kunming, China; ^4^ Shanghai Jizhi Biotechnology Corporation, Shanghai, China

**Keywords:** *Whitmania* species, chromosome-level genome, antithrombotic gene, genetic variation, gene expression

## Abstract

Leeches are well known for blood-feeding habits and are widely used for medicinal purposes as they secrete various antithrombotic substances. However, some leeches exhibit non-hematophagous habits and their significance for medicinal use is controversial. Here we provide the chromosome-level genomes of two non-hematophagous leeches, *Whitmania acranulata* and *Whitmania laevis*, and, in combination with previous results from *Whitmania pigra*, we compared these genomes with an emphasis on antithrombotic biomolecules. All three species had the same chromosome number of 11. The genome size, repeat site percentage, and number of protein-coding genes of *W. laevis* (173.87 Mb, 28.28%, 23,818) were similar to those of *W. pigra* (169.37 Mb, 27.02%, 24,156), whereas these values of *W. acranulata* (181.72 Mb, 29.55%, 27,069) were higher than those of the other two leeches. *W. laevis* was a monophyletic clade of *W. pigra*, whereas *W. acranulata* had a paraphyletic relationship with *W. pigra*. The number of antithrombotic genes in *W. laevis* (*N* = 76) was similar to that of *W. pigra* (*N* = 79), whereas *W. acranulata* (*N* = 102) had apparently more such genes. Of the 21 gene families, 9 and 11 were differentially expressed in *W. acranulata* and *W. laevis* compared to *W. pigra*, respectively. The expression profiles of the antithrombotic gene families were more similar between *W. acranulata* and *W. laevis*. Although there were several cases of gene loss or pseudogenization, most antithrombotic genes of the three *Whitmania* species were intact and transcribable. These results provide valuable insights into the evolution of non-hematophagous leeches and development of antithrombotic drugs.

## 1 Introduction

Leeches, which belong to the class Hirudinea, are renowned for their blood-sucking habits and are found all over the world except Antarctica (Sawyer, 1986). Many leeches target the blood of vertebrates and release an array of antithrombotic biomolecules, including anticoagulants, antiplatelet agents, fibrinolysis enhancers, and tissue penetration enhancers, which are essential to overcome the host’s natural hemostatic mechanisms (Sig et al., 2017). Dozens of such biomolecules involving more than 20 protein families (e.g., hirudin, antistasin, guamerin, eglin, bdellin, saratin, destabilase, and hyaluronidase) have been identified in various leech species (Babenko et al., 2020; Kvist et al., 2020; Liu et al., 2023; Liu et al., 2024; Zhao et al., 2024). Some biomolecules have played an important role in medical and pharmaceutical developments. For example, the derivatives/analogues (lepirudin, desirudin, and bivalirudin) of hirudin, which is the most potent natural thrombin inhibitor found to date, have been available for clinical use for decades (Warkentin, 2004). In addition, antistasin, the first Factor Xa inhibitor, has inspired the development of several well-known anticoagulants such as rivaroxaban and otamixaban (Perzborn et al., 2011).

Although sanguivory is the most prominent aspect of leech behavior in certain species, other feeding modes are common in the remaining species (Borda and Siddall, 2004). Of note is the genus *Whitmania*, which is the sister clade of the genus *Hirudo* (Phillips and Siddall, 2009; Zhao et al., 2021). At least three species of *Whitmania* have been found: *Whitmania pigra* (Whitman, 1884), *Whitmania laevis* (Baird, 1869), and *Whitmania acranulata* (Whitman, 1886). *W. pigra* is a large species of leech, typically 60–130 mm long and 13–20 mm wide. The maximum body length and body width could reach to 250 and 40 mm, respectively (Yang, 1996), with body weight over 50 g. *W. pigra* are specific predators of snails, although they have retained tooth plates in their jaws, the blood-feeding ability have been lost. *W. laevis* have a median body size, typically 32–81 mm long and 5–12 mm wide. They have a broader diet, including snails and insect larvae, and interestingly, no tooth plates are detected in *W. laevis*, suggesting that the blood-sucking trait is more thoroughly degraded in *W. laevis* than in *W. pigra*. (Qiao et al., 2013). *W. acranulata* have a small body size, only 28–67 mm long and 3.5–8 mm wide. Although *W. acranulata* retain tooth plates in their jaws, they are swallowers and feed on aquatic earthworms and insect larvae (Yang, 1996).

According to the Pharmacopoeia of the People’s Republic of China (PPRC) (Commission, 2020), which is an integral part of China’s drug laws and regulations, three species of leeches (*H. nipponia*, *W. pigra*, and *W. acranulata*) have been considered as legal materials for “Shuizhi” products. For the hematophagous *H. nipponia*, scientists are convinced of its medicinal value, while for *W. pigra* and *W. acranulata*, there has been a long-standing debate as to whether they can serve as the basic source of “Shuizhi” due to their non-blood-sucking habits (He et al., 2021). Some researchers argue that the two *Whitmania* species are no longer valid “medicinal leeches” because they have completely lost their blood-sucking behavior. As a result, their antithrombotic capabilities were likely lost during their habit change and should therefore be excluded from the PPRC (Song and Zhou, 1997). Conversely, other researchers argue that these two species should be included in the PPRC due to their close relationship with the genus *Hirudo* (Phillips and Siddall, 2009; Zhao et al., 2021). They believe that most of the antithrombotic proteins remain active. The latter view seems to be more accurate, since the anticoagulant properties (Ding et al., 2016; Zhang et al., 2020) and antiplatelet aggregation capabilities (Li et al., 1997; Wang et al., 2019) have been repeatedly confirmed in the two *Whitmania* species. Recent studies support that at least one type of hirudin from *W. pigra* has anticoagulant activity (Müller et al., 2022). It should be noted that, in the Medicinal Fauna of China (Li et al., 2013), another authoritative publication on Chinese traditional medicine, *W. laevis* is also considered as a legal material for “Shuizhi” products. A previous study showed that this species also possesses some anticoagulant and antiplatelet activates (Li et al., 1997).

With the rapid development of high-throughput sequencing technology, genomes of several medicinal leeches have been described: *Hirudo medicinalis* (Babenko et al., 2020; Kvist et al., 2020), *Hirudinaria manillensis* (Guan et al., 2019; Zheng et al., 2022), *W. pigra* (Tong et al., 2022; Zheng et al., 2022; Liu et al., 2024), and *Hirudo nipponia* and *Hirudo tianjinensis* (Zhao et al., 2024). Interestingly, a total of 79 antithrombotic genes were identified from the hematophagous *W. pigra*, even more than the typical blood-feeding *H. manillensis*, which had 72 antithrombotic genes (Liu et al., 2023). Combined with RNA-Seq-based gene expression analyses, our recent study showed that the number and expression level of antithrombotic genes of a non-hematophagous leech are not always lower than those of a hematophagous leech (Liu et al., 2024). The unique life history of non-hematophagous leech species provides an excellent opportunity to understand the evolution of antithrombotic-related genes in leeches. Here, we provide a chromosome-scale genome of *W. acranulata* and *W. laevis* from which we identified potential antithrombotic genes of the two species. Furthermore, in combination with RNA-Seq data, we calculated the expression levels of the antithrombotic genes. Combined with our previous results from *W. pigra* (Liu et al., 2024) we aim to systematically compare the compositions and expression of antithrombotic genes among the three non-hematophagous *Whitmania* leeches.

## 2 Materials and methods

### 2.1 Sequencing, assembling, and gene prediction

*W. acranulata* and *W. laevis* samples were collected in Yutai County, Shandong, China (E 116°28′55″, N 35°4′17″). After removal of the digestive tracts, total genomic DNA or RNA was isolated from fresh anterior body tissues. Similar as our recent study on *H. nipponia* and *H. tianjinensis* (Zhao et al., 2024), one Nanopore library was constructed respectively for *W. acranulata* and *W. laevis* and was sequenced using an ONT sequencer (Oxford Nanopore Technologies, Oxford, UK). At the meantime, one Hi-C, one Survey, and three RNA-Seq libraries for *W. acranulata* and *W. laevis* were constructed and were sequenced using the Illumina HiSeq 2000 sequencing platform (Illumina Inc., San Diego, CA, United States). Four individuals were used for each species: one for the Nanopore, Hi-C and Survey libraries, and the other three for each of the three RNA-Seq libraries.

The genome was assembled with ONT reads using NextDenovo v2.5.0 (parameters: read_type = ont, genome_size = 200 m, sort_options = -m 50 g -t 30, and also other default settings) (Hu et al., 2024), refined using NextPolish v1.4.0 (Hu et al., 2020) with Survey reads, and were then integrated with Hi-C reads using YaHS v1.1a (Zhou et al., 2023). The mitochondrial genome was assembled with Survey reads using GetOrganelle v1.7.7.0 with “-F animal_mt” and other default settings (Jin et al., 2020). BUSCO v4.1.4 (with the eukaryota_odb10 database) (Seppey et al., 2019) and Merqury v1.3 (Rhie et al., 2020) were used to assess the thoroughness and quality of the genomes, respectively. RepeatMasker v4.1.2-pl (Flynn et al., 2020) were used to search for repetitive sequences, and the repeat-masked scaffolds were used for gene prediction using a BRAKER-plus strategy (Liu et al., 2023).

### 2.2 Phylogenomic and chromosome syntenic analysis

Combined with previously reported genome of *W. pigra* (Liu et al., 2024), *H. medicinalis* (Kvist et al., 2020), *H. manillensis* (Liu et al., 2023), *H. nipponia* and *H. tianjinensis* (Zhao et al., 2024), and *Dinobdella ferox* (Gao et al., 2023), we tested the phylogenetic relationship among the three *Whitmania* species. The orthologs of the coding sequences (CDS) of all species were detected using OrthoFinder v2.3.11 with “-S diamond” and other default settings (Emms and Kelly, 2019), aligned using MACSE (Ranwez et al., 2011), and were concatenated using Seqkit v0.10.2 (Shen et al., 2016). IQ-TREE (Nguyen et al., 2015) was used to reconstruct trees using 1,000 bootstrap replicates (*Dinobdella ferox* was set as the outgroup).

We also used NGenomeSyn v1.41 (He et al., 2023) to visualize microcolinearity between chromosomes of the three *Whitmania* genomes. First, pairwise comparisons were performed using the GetTwoGenomeSyn.pl scripts with parameters set as “-MinLenA 5,000,000, -MinLenB 5,000,000, -MinAlnLen 5000, -MappingBin minimap2”. The results of the pairwise comparisons were then integrated using the main program NGenomeSyn.

### 2.3 Expression analysis of antithrombotic genes

The relative expression levels of antithrombotic genes of the three *Whitmania* species were estimated using the RNA-Seq reads. The total predicted CDS of each species were used as mapping references. The TPM values of antithrombotic genes were calculated using salmon v1.0.0 with a kmer size of 31 (Patro et al., 2017). Finally, the total TPM of all members within each antithrombotic gene family was calculated for interspecies comparison.

The TPM of each gene family of *W. acranulata* vs. *W. pigra* and *W. laevis* vs. *W. pigra* were compared using the non-parametric Mann-Whitney *U* test in SPSS v25.0 (IBM Corp., Armonk, NY, United States). In addition, to show the overall similarity of antithrombotic gene expression patterns among the three *Whitmania* species, the gene expression spectra of all samples were clustered using hierarchical cluster analysis (the TPM values of each sample were prior rescaled to the interval [0, 1]) in the R package pheatmap v1.0.12. The use of the above software or programs, if not specified, means that they have been run with default settings and parameters.

## 3 Results

### 3.1 Basic information of genome assembly

**FIGURE 1:**
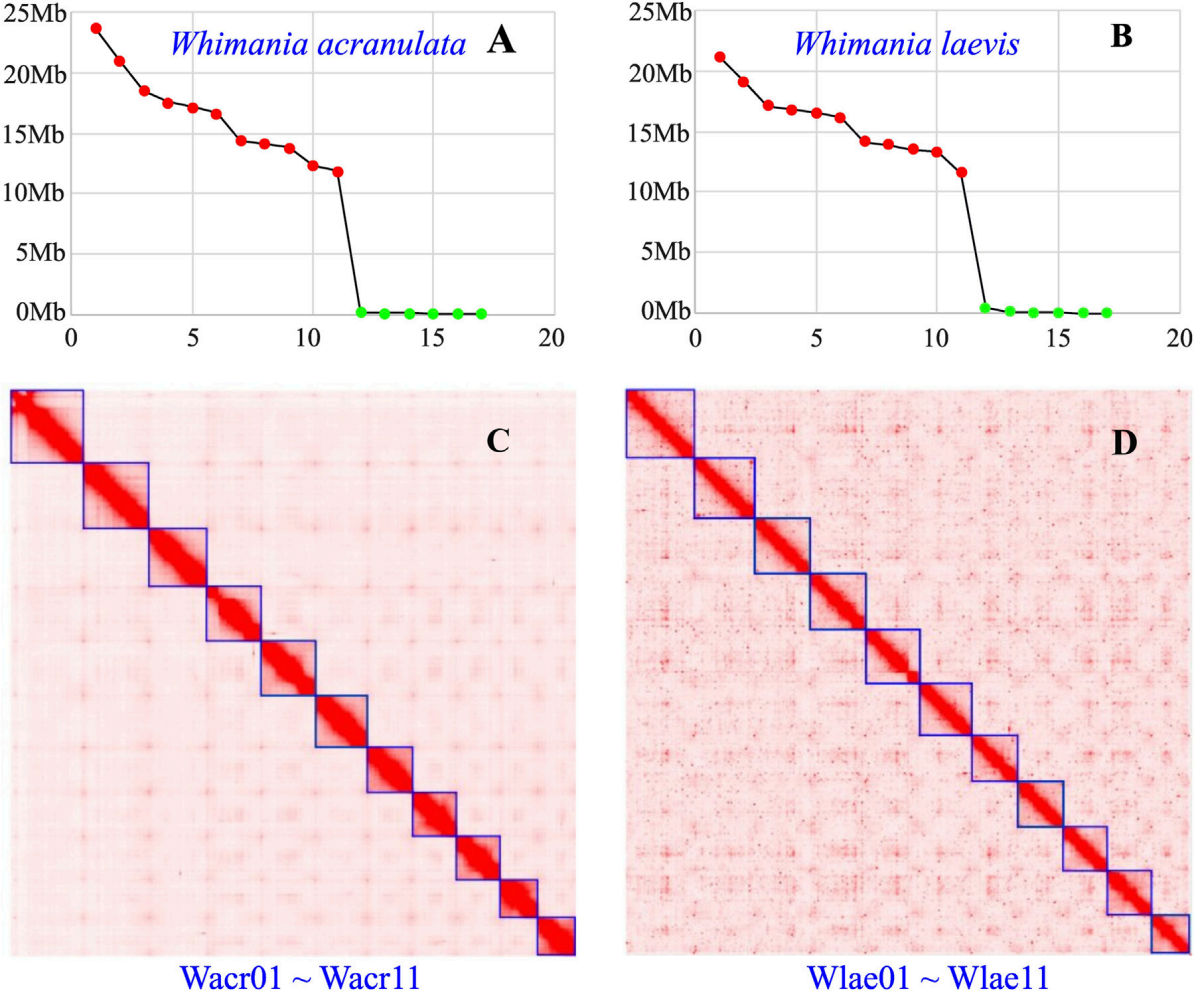
Assembly information of *Whitmania acranulata* and *Whitmania laevis* genomes. Scaffold 187 length distribution of **(A)** *W. acranulata* and **(B)** *W. laevis* (the red dots indicate the 11 chromosomes; the green dots indicate the remaining short scaffolds). Hi-C links among the chromosomes of **(C)** *W. acranulata* and **(D)** *W. laevis* (darker red color indicates higher contact probability; Wacr and Wlae are abbreviations of *W. acranulata* and *W. laevis*, respectively; the lengths of the chromosomes Wacr01 ∼ Wacr11 and Wlae01 ∼ Wlae11 decrease sequentially).

After assembly and polishing, 45 and 88 contigs with N50 of 9.40 and 11.76 Mb were obtained for *W. acranulata* and *W. laevis*, respectively. Coincidentally, 17 scaffolds with a total length of 181.72 and 173.87 Mb were obtained for both the *W. acranulata* and *W. laevis* genome assemblies after consolidation by Hi-C reads. For both species, the length of the first 11 longest scaffolds and the remaining 6 scaffolds showed a highly discontinuous distribution (Figures 1A, B). The well-resolved Hi-C maps further indicated that the 11 longest scaffolds are predicted chromosomes (Figures 1C, D), resulting in a chromosomal anchoring rate over 99.5%. In addition, a circular complete mitochondrial genome was obtained for both species (Table 1).

**TABLE 1:** Basic information of genomes and protein-coding genes of *Whitmania acranulata*, *Whitmania laevis*, *Whitmania pigra* (Liu et al., 2024).

Item	*W*. *acranulata*	*W*. *laevis*	*W*. *pigra*
Number of contigs	45	88	62
N50 of contigs (Mb)	9.40	11.76	9.88
Number of chromosomes	11	11	11
Total genome size (Mb)	181.73	173.88	169.37
Mitogenome length (bp)	14,505	17,499	15,985
Number of protein-coding genes	22,810	21,347	21,947

The genomes of *W. acranulata* and *W. laevis* are available in Supplementary Files S1 and S2, respectively. As shown in Table 1, the three *Whitmania* species had the same number of chromosomes (*N* = 11). The genome size of *W. acranulata* were a bit larger than those of *W. laevis* and *W. pigra*. In contrast, the mitogenome length of *W. acranulata* was smaller than those of *W. laevis* and *W. pigra*.

### 3.2 Genome quality and repeat sequences

The BUSCO completeness analysis showed that, for *W. acranulata*, 250 of the 255 BUSCOs were captured, while 5 (2.0%) BUSCOs were missed. For *W. laevis*, 248 BUSCOs were captured, while 7 (2.8%) BUSCOs were missed. We also used Merqury (Rhie et al., 2020) to assess the quality of our genome assembly and obtained quality value scores of 36.54 and 39.92 for the *W. acranulata* and *W. laevis* genomes, respectively.

We searched the genomes for repeat sequences using RepeatMasker (Flynn et al., 2020). A total of 29.55% sites were identified as repeats in the *W. acranulata* genome: Retroelements had the highest percentage, followed by Unclassified repeats and DNA transposons. As to *W. laevis*, a total of 28.28% of the sites were identified as repeats: Unclassified repeats had the highest percentage, followed by Unclassified repeats and DNA transposons. The total percentage of repeat sites in *W. acranulata* was higher than those in *W. laevis* and *W. pigra* (Liu et al., 2024), mainly due to the apparently higher percentage of Retroelements in *W. acranulata* (Table 2).

**TABLE 2:** Percentage of different repeat sequence types in the three *Whitmania* genomes.

Item	*W*. *acranulata*	*W*. *laevis*	*W*. *pigra*
Retroelements	12.54	9.77	7.82
DNA transposons	5.17	6.82	6.80
Rolling-circles	0.14	0.22	0.24
Unclassified	9.75	10.66	9.46
Small RNA	0.00	0.00	0.00
Satellites	0.00	0.15	0.00
Simple repeats	1.95	0.66	2.70
Low complexity	0.00	0.00	0.00
Total	29.55	28.28	27.02

### 3.3 Phylogenomics and chromosome syntenic analyses

**FIGURE 2:**
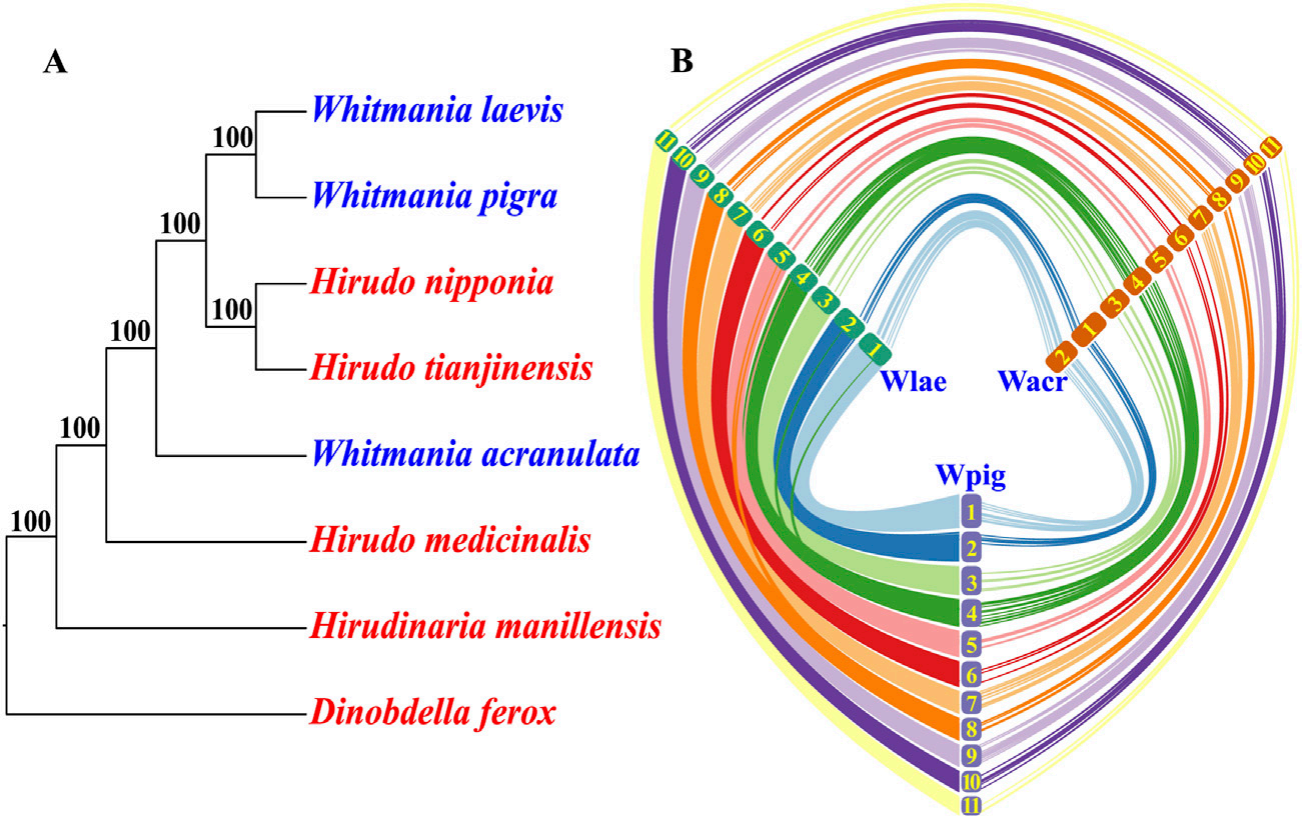
Genomic relationships among the three *Whitmania* leeches. **(A)** Phylogenomics of the three *Whitmania* species as well as other related leech taxa (*Dinobdella ferox* has been set as the 12 outgroup); **(B)** Chromosome syntenic relationships among the three *Whitmania* species (more curves indicate higher collinearity levels; the numbers indicate the chromosomes of each species; larger numbers represent smaller chromosomes).

A total of 4,493 orthologs were identified among the eight leech species. Phylogenetic analysis based on the concatenated sequences (5,335,974 bp in length) produced a highly confident consensus tree, i.e., all of the nodes had a bootstrap percentage of 100%. Unexpectedly, the three *Whitmania* species did not form into a monophyletic group. Instead, *W. pigra* and *W. laevis* formed into a subclade that was more closely related to a *Hirudo* subclade (*H. nipponia* and *H. tianjinensis*) than to *W. acranulata* (Figure 2A).

Chromosome syntenic analysis showed that the chromosomes of *W. pigra* and *W. laevis* were perfectly matched. The chromosome length order is identical and there were massive syntenic segments between the two species. In contrast, there was an inconsistency in chromosome length order between *W. acranulata* and the other two *Whitmania* species, i.e., the longest chromosome (Wacr01) of *W. acranulata* corresponded to the second longest chromosome of *W. pigra* (Wpig02) and *W. laevis* (Wlae02). Furthermore, the syntenic segments between the *W. acranulata* and the other two *Whitmania* species were obviously fewer than those between *W. pigra* and *W. laevis* (Figure 2B).

### 3.4 Composition of antithrombotic genes

Based on the BRAKER-plus strategy, 22,810 and 21,347 protein-coding genes were predicted for *W. acranulata* and *W. laevis*, respectively. The number of protein-coding genes in *W. laevis* and *W. pigra* were similar, but were smaller than those in *W. acranulata* (Table 1). The GFF files and all predicted CDS (including isoforms) of the two species (Supplementary Files S3–S6) were available as Supplementary Material. A total of 102 and 76 antithrombotic genes were identified from the genomes of *W. acranulata* and *W. laevis*, respectively. According to our previous studies (Liu et al., 2023; Liu et al., 2024), these genes could be classified into different gene families (Table 3; for more information on the functions of these protein families, see also the two previous papers and the references therein).

**TABLE 3:** Gene numbers of the antithrombotic gene families of the leeches.

Gene family	*W. acranulata*	*W. laevis*	*W. pigra*	Function
*hirudin*	0	0	7	coagulation inhibitor
*progranulin*	1	1	1	coagulation inhibitor
*antistasin*	3	3	2	coagulation inhibitor
*lefaxin*	3	3	3	coagulation inhibitor
*therostasin*	1	1^#^	1	coagulation inhibitor
*hirustasin*	1	2	1	coagulation inhibitor
*hirustasin-like*	8	8	9	coagulation inhibitor
*guamerin*	1	1	1	coagulation inhibitor
*piguamerin*	3	1	1^#^	coagulation inhibitor
*bdellastasin*	1	1	1	coagulation inhibitor
*eglin*	11	5	2	coagulation inhibitor
*bdellin*	3	2	1	coagulation inhibitor
*LDTI*	6	1	1	coagulation inhibitor
*HMEI*	35^##^	21^#^	20^#^	coagulation inhibitor
*saratin*	0	6^#^	11^#^	platelet aggregation inhibitor
*apyrase*	9	4	3	platelet aggregation inhibitor
*lumbrokinase*	2	4	4	platelet aggregation inhibitor
*destabilase*	6	6	4	fibrinolysis enhancer
*GGT*	4	2	2^#^	fibrinolysis enhancer
*LCI*	1	1	1	fibrinolysis enhancer
*hyaluronidase*	3	3	3	tissue penetration enhancer
total	102	76	79	—

*LDTI*, leech-derived tryptase inhibitor; *HMEI*, *Hirudinaria manillensis* elastase inhibitor; *GGT*, gamma-glutamyl transpeptidase; *LCI*, leech carboxypeptidase inhibitor; ^#^, including one pseudogene; ^##^, including two pseudogenes; —, Not applicable.

In contrast to *W. pigra*, which had two thrombin inhibitor families (*hirudin* and *progranulin*), only the *progranulin* was identified in *W. acranulata* and *W. laevis*, while the *hirudin* family was completely lost in the latter two species. Three factor Xa inhibitor families (*antistasin*, *lefaxin*, and *therostasin*) were recovered in both *W. acranulata* and *W. laevis*. As in *W. pigra*, most of the remaining coagulation inhibitors (*hirustasin*, *hirustasin-like*, *guamerin*, *piguamerin*, *bdellastasin*, *eglin*, *bdellin*, *LDTI*, and *HMEI*) were recovered in both *W. acranulata* and *W. laevis*. Of the three antiplatelet families (*saratin*, *apyrase*, and *lumbrokinase*), two and three families were recovered in *W. acranulata* and *W. laevis*, respectively. Finally, three families of fibrinolysis enhancers (*lumbrokinase*, *destabilase*, *GGT*, and *LCI*) and one family of tissue penetration enhancers (hyaluronidase) were also recovered in both *W. acranulata* and *W. laevis* (Table 3).

### 3.5 Variation of antithrombotic genes/proteins

**FIGURE 3:**
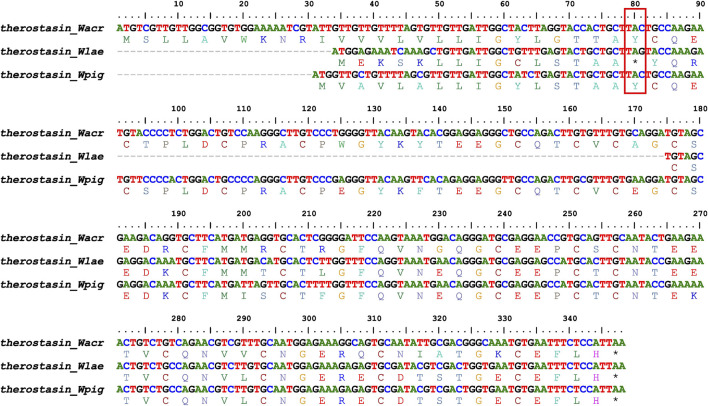
Alignment of therostasins and their coding sequences (red frame, early terminal codon of *W. laevis* therostasin).

There was massive genetic variation among the members in each of the antithrombotic gene/protein families (Supplementary Figures S1–S17), including several pseudogenization cases (Table 2). Although hirudin is the first identified and most representative antithrombotic bioactive protein in leeches (Krezel et al., 1994), their coding genes were completely lost from the genomes of *W. acranulata* and *W. laevis* (Supplementary Figure S1), making it impossible to show their interspecific genetic variation. One or more members of the gene families *therostasin* (*therostasin_Wlae*, Supplementary Figure S5, Figure 3), *piguamerin* (*piguamerin_Wpig*, Supplementary Figure S6), *HMEI* (*HMEI_Wacr25*, *HMEI_Wacr28*, *HMEI_Wpig07*; Supplementary Figure S10), *saratin* (*saratin_Wlae4* and *saratin_Wpig03*, Supplementary Figure S11), and *GGT* (*GGT_Wpig2*, Supplementary Figure S15) were pseudogenized.

There were many proteins whose reactive residues were predicted by functional verification tests. The arginine from the C**R**EHC segment was predicted to be the catalytic residue in the archetypal antistasin (Dunwiddie et al., 1989), but of the eight proteins from the three *Whitmania* species, only one (antistasin_Wpig2) was conserved at this site (Supplementary Figure S3). The predicted catalytic residue arginine (C**R**IYC) in the archetypal therostasin (Chopin et al., 2000) was replaced by phenylalanine in all therostasins from the three *Whitmania* species (Supplementary Figure S5). The catalytic arginine (C**R**IRC) of the archetypal hirustasin (Mittl et al., 1997) was conserved in the hirustasins of *W. pigra*, but not in those of the other species (Supplementary Figure S6). The catalytic arginine (C**R**KYC) of the archetypal piguamerin (Kim et al., 2001) was conserved in all piguamerins except piguamerin_Wlae (Supplementary Figure S6). The catalytic methionine (C**M**IFC) of the archetypal guamerin (Jung et al., 1995) and the catalytic arginine (C**K**VKC) of the archetypal bdellastasin (Moser et al., 1998) were conserved in all guamerins and bdellastasins from the three *Whitmania* species (Supplementary Figure S6).

The reactive residues leucine and asparagine (McPhalen et al., 1985) in the archetypal eglin (T**LD**LR) were conserved in 7 of 15 eglins from the three *Whitmania* species (Supplementary Figure S7). The reactive residue lysine (CT**K**EL) in the archetypal bdellin (Kim et al., 2001) was conserved in three of five bdellins from the *Whitmania* species (Supplementary Figure S8). The reactive residues lysine and isoleucine (P**KI**LK) of the archetypal LDTI (Sommerhoff et al., 1994) were conserved in the LDTIs of *W. laevis* and *W. pigra*, but not those of *W. acranulata* (Supplementary Figure S9). The catalytic histidine (KI**H**NM) of the archetypal destabilase (Marin et al., 2023) was conserved in all destabilases from the three *Whitmania* species, except for destabilase_Wlae5 (Supplementary Figure S14). Finally, the catalytic threonine (HG**T**AH) of the archetypal GGT (Castellano and Merlino, 2012) was conserved in all GGTs from the three *Whitmania* species (Supplementary Figure S15).

### 3.6 Expression of antithrombotic genes

**FIGURE 4:**
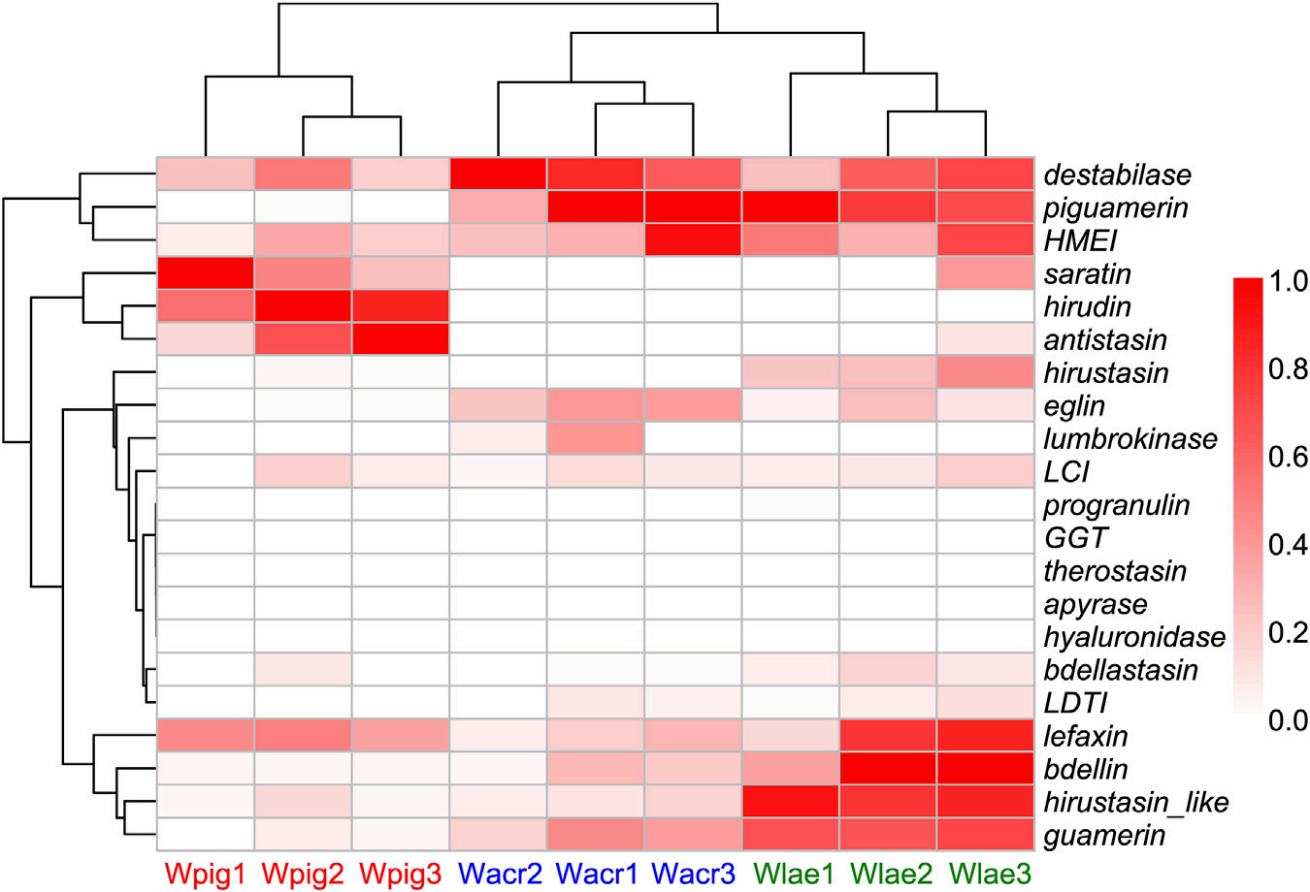
Heatmap of the expression level of antithrombotic genes of the three *Whitmania* species The TPM (transcripts per million) values of each sample was rescaled to the interval [0, 1]; darker red color indicates higher expression level. *W. acranulata*: Wacr1 ∼ 3, SRA No. SRR27841062 ∼ SRR27841060; *W. laevis*: SRA No. Wlae1 ∼ 3, SRR26541749 ∼ SRR26541747; *W. pigra*: Wpig1 ∼ 3, SRA No. SRR26541745 ∼ SRR26541743).

Based on the RNA-Seq data sequenced in this study we calculated the total TPM (transcripts per million) of each antithrombotic gene family of *W. acranulata* and *W. laevis*. Combined with previously reported data of *W. pigra*, we performed pairwise comparisons on the TPM of each gene family among the three species using the non-parametric Mann-Whitney *U* test. Of the 21 gene families, 9 were significantly differentiated between *W. acranulata* and *W. pigra*, and 11 were significantly differentiated between *W. laevis* and *W. pigra* (Table 4). The gene family *antistasin* of both *W. acranulata* and *W. laevis* had a lower express level than *W. pigra*. In contrast, the gene families *guamerin*, *piguamerin*, *eglin*, and *LDTI* of *W. acranulata* and *W. laevis* had higher expression levels than *W. pigra*. Hierarchical cluster analysis showed that the samples were grouped into three clusters, each corresponding to one of the three *Whitmania* species. The *W. acranulata* and *W. laevis* samples had closer expression patterns, while the expression patterns of the *W. pigra* samples were less similar to the other two species (Figure 4).

**TABLE 4:** The total TPM values (Mean ± SD) of each gene family.

Gene family	*W. acranulata*	*W. laevis*	*W. pigra*
*hirudin*	0.0 ± 0.0	0.0 ± 0.0	11,929.3 ± 9,848.5
*progranulin*	24.9 ± 20.3*	147.6 ± 121.4	61.3 ± 9.5
*antistasin*	163.8 ± 72.5*	663.4 ± 1,064.3*	11,459.8 ± 9,897.5
*lefaxin*	4,359.6 ± 1,049.1*	10,879.8 ± 6,651.4	8,970.7 ± 1,647.5
*therostasin*	0.1 ± 0.0	0.5 ± 0.9	79.6 ± 31.5
*hirustasin*	125.6 ± 29.8	5,819.8 ± 1958.3*	702.3 ± 570.1
*hirustasin-like*	2,978.1 ± 1,346.0	16,550.6 ± 3,417.3*	2,157.3 ± 1,260.2
*guamerin*	8,833.9 ± 3,894.1*	13,255.2 ± 1,537.3*	1,485.1 ± 855.7
*piguamerin*	18,474.4 ± 4,611.1*	16,059.8 ± 5,356.2*	251.2 ± 149.0
*bdellastasin*	255.1 ± 45.1	2,121.7 ± 905.6*	725.3 ± 626.9
*eglin*	9,791.0 ± 6,286.7*	2,603.9 ± 1759.3*	323.9 ± 100.3
*bdellin*	3,152.0 ± 1,592.9	14,618.0 ± 5,658.2*	620.0 ± 165.5
*LDTI*	884.4 ± 705.3*	1,305.3 ± 1,005.2*	63.4 ± 48.6
*HMEI*	12,434.4 ± 6,605.3	9,890.5 ± 3,860.0	5,608.7 ± 1,492.1
*saratin*	0.0 ± 0.0	2,327.9 ± 4,026.1	4,580.8 ± 4,007.4
*apyrase*	61.7 ± 14.5	24.5 ± 7.9	12.9 ± 6.7
*lumbrokinase*	4,061.2 ± 3,625.9	42.0 ± 5.5*	15.2 ± 9.9
*destabilase*	32,661.6 ± 36,165.4	9,928.1 ± 3,736.7	8,672.2 ± 4,262.8
*GGT*	46.8 ± 9.1	94.0 ± 40.0	159.5 ± 67.4
*LCI*	1724.3 ± 585.2	2,169.5 ± 1,040.4	2,889.3 ± 1,157.1
*hyaluronidase*	29.9 ± 10.6	77.2 ± 59.0	50.7 ± 12.2

*Significant differences of *Whitmania acranulata* vs. *Whitmania pigra* and *Whitmania laevis* vs. *Whitmania pigra* at the level of *P* < 0.05.

## 4 Discussion

### 4.1 General information of the *Whitmania* genomes

A high-quality genome is essential for molecular evolutionary analyses of organisms. Recent high-throughput sequencing and assembly technologies make it easier to obtain a chromosome-scale genome. Here, we used the third- and the next-generation sequencing methods to obtain chromosome-scale genomes of *W. acranulata* and *W. laevis*. For both species the predicted chromosomes constitute over 99.5% of the total scaffold length, indicating that the genome assemblies have high sequence continuity. The BUSCO analyses showed that over 97% of the BUSCOs were captured in the two genomes, indicating their high completeness. The Merqury analyses on the two genomes yielded quality scores above 36, also higher than the previous study on other leech species (Zheng et al., 2022). In addition, we assembled a complete circular mitochondrial genome for each of the two leeches. As a result, based on the above parameters, we have for the first time provided high quality and nearly complete genomes of the two non-hematophagous leeches.

By comparing the genome of *W. pigra* obtained in our previous studies (Liu et al., 2024), we showed the similarities and differences among the three non-hematophagous *Whitmania* genomes. Although the three species had the same chromosome numbers (*N* = 11), there were several substantial differences on the genomic characteristics between *W. acranulata* and the other two species. First, the genome size, repeat site percentage, and protein-coding gene number of *W. acranulata* were larger than those of *W. laevis* and *W. pigra*. Second, the chromosome syntenic analysis showed a well genome collinearity between *W. laevis* and *W. pigra*, however, the collinearity between *W. acranulata* and the other two *Whitmania* species was apparently weakened. Third, the phylogenetic analysis showed that the three *Whitmania* species formed into a paraphyletic group, i.e., *H. nipponia* and *H. tianjinensis*, rather than *W. acranulata*, became a sister clade of *W. pigra* and *W. laevis*. A previous study has found that the members of family Haemopidae (*Haemopis* spp. and *W. laevis*) were found not to be monophyletic (Phillips and Siddall, 2009). Interestingly, the present study further demonstrated the complexity of relationships among members of the Haemopidae family: even members of with the single *Whitmania* genus could be non-monophyletic.

### 4.2 Antithrombotic genes and their corresponding proteins

The composition of antithrombotic genes was also different among the three *Whitmania* species. *W. acranulata* had 102 antithrombotic genes, much more than *W. laevis* (*N* = 76) and *W. pigra* (*N* = 79). Our previous studies found that the four hematophagous leeches *H*. *manillensis H*. *nipponia*, *H*. *tianjinensis* and *H. medicinalis* had 72, 86, 83 and 74 antithrombotic proteins, respectively (Liu et al., 2023; Zhao et al., 2024). These results showed that there is no significant difference between non-hematophagous and hematophagous leeches, at least in the number of antithrombotic genes. Although the molecular biology of non-hematophagous leeches has received much less attention than that of hematophagous leeches, at least three proteins from *W. pigra*, i.e., lumbrokinase (inferred from sequence comparison) (Jiang et al., 2020), hirudin (Müller et al., 2022) and destabilase (Cheng et al., 2025), have been shown to have antithrombotic activity, suggesting that non-haemophagous leeches also have great potential for medicinal use. There were 35 *HMEIs* in *W. acranulata*, also much more than in *W. laevis* (*N* = 21) and *W. pigra* (*N* = 20). In the *eglin* family, 11 were found in *W. acranulata*, while only five and two were found in *W. laevis* and *W. pigra*, respectively. Gene loss events seem irregularly distributed in the three *Whitmania* species, e.g., seven *hirudins* were found in *W. pigra*, but this gene family disappeared completely in *W. acranulata* and *W. laevis*. Similarly, 11 and six *saratins* were identified in *W. pigra* and *W. laevis*, respectively, whereas this gene family was completely undetectable in the *W. acranulata* genome. Furthermore, there were a lot of genetic variations among the members of each gene/protein family, For example, of the 11 eglins found in *W. acranulata*, no two proteins have the same sequence. These results indicate that the antithrombotic biomolecules in *Whitmania* leeches are extremely variable, while what causes these species to have such high genetic instability deserves further investigation in the future.

The hierarchical cluster analysis based on the TPM of the antithrombotic families showed that the samples from each species perfectly formed their respective clusters, indicating that the expression profiles of these gene families were apparently different among these *Whitmania* species. In contrast to the results of the phylogeny and chromosome synteny analyses, the antithrombotic gene expression profile showed that *W. laevis* was more closely related to *W. acranulata* than to *W. pigra*. Looking specifically at each gene family, there were many differences in TPM levels among *Whitmania* species. For example, of the 21 gene families, 9 and 11 were differentially expressed in *W. acranulata* and *W. laevis* compared to *W. pigra*, respectively. These results suggest that different species have evolved different expression regulation of these genes for different survival strategies. It should be noted that (as kindly suggested by the anonymous reviewers) differences in antithrombotic gene expression between species may not fully reflect differences in their medicinal activity. Firstly, the starting materials for the present study were collected in the wild and many environmental and biotic factors such as season, age or nutritional status of the leeches may influence gene expression. Secondly, it is not the genes, but the respective encoded protein, that determine the final medicinal values. Next, we will use proteomics and recombinant protein synthesis technology to assess the protein abundances and activities of these differentially expressed genes to further measure the contribution of these genes to the medicinal value of the three non-haemophagous leeches.

### 4.3 Perspectives on pharmacological application

Leeches have been used as a medical and pharmaceutical resource for many centuries (Michalsen et al., 2007). Although *Whitmania* leeches cannot be used for leech therapy due to their non-hematophagous nature, these species still have pharmaceutical value in the treatment of thrombosis diseases (Commission, 2020; Yu et al., 2022). *W. pigra*, which has the largest body size, the most abundant resources, and is relatively easy to cultivate, has become the primary material for traditional Chinese medicine “Shuizhi” (Liu and Yang, 2014). In contrast, *W. acranulata* and *W. laevis* were less concerned due to the scarcity of resources. It has been repeatedly confirmed that, all the three *Whitmania* species mentioned in this study have anticoagulant and/or antiplatelet activities (Ou et al., 1996; Li et al., 1997; Guan et al., 2012; Zhang et al., 2012; Li et al., 2014; Ding et al., 2016; Cheng, 2018; Wang et al., 2019; Zhang et al., 2020; Zhong et al., 2020). However, probably due to the complexity of the composition and expression of the different antithrombotic genes, there were inconsistencies in different studies on the antithrombotic activities of the *Whitmania* species. For example, a previous study showed that *W. pigra* and *W. acranulata* had similar antithrombin activities (Wang et al., 2013), while some other studies indicated that the antithrombin activities of *W. pigra* were higher than *W. acranulata* (Cheng, 2018), or conversely (Zhang et al., 2012; Zhang et al., 2020). The large number of antithrombotic genes identified in this study, and the availability of complete CDSs for most of these genes (except for pseudogenes), will provide opportunities to test their function by producing recombinant proteins (Gupta et al., 2016) and performing *in vitro* (Liu et al., 2023) and *in vivo* (Tang et al., 2018) experiments.

Hirudin is the most potent thrombin-specific inhibitor identified to date and is a representative pharmacologically active substance in leeches (Markwardt, 2002; Chen et al., 2021). In the PPRC, antithrombin activity is the only standard for determining the quality of “Shuizhi” (Commission, 2020). It was not surprising that the extracts of *W. pigra* samples had antithrombin activities, as there were seven hirudins, of which at least one was shown to be functionally active (Müller et al., 2022). Although no *hirudin* was found in their genomes, *W. acranulata* (Li et al., 1997; Wang et al., 2013) and *W. laevis* (Li et al., 1997) were reported to have antithrombin activities, probably due to the antithrombin activity of other proteins such as the granulins (Hong and Kang, 1999). The current version of the PPRC listed *W. pigra* and *W. acranulata*, but not *W. laevis*, as a legal drug material. However, since there were still dozens of antithrombotic genes in *W. laevis*, and most importantly, there were 11 genes with higher expression levels than in *W. pigra*, we suggest that this species may also have application values for antithrombotic drug development, although further studies are still needed.

## 5 Conclusion

In summary, we provide two nearly complete high-quality genomes of *W. acranulata* and *W. laevis*. The genome size, repeat site percentage, and number of protein-coding genes of *W. laevis* were similar to those of *W. pigra*, whereas these indices of *W. acranulata* were higher than those of the other two leeches. Both the compositions and the expression profiles of antithrombotic genes were apparently different among the three leeches.

## Data Availability

The data presented in the study are deposited in the figshare repository, accession: https://doi.org/10.6084/m9.figshare.25757583. The raw data from our genome project was deposited in the SRA (Sequence Read Archive) database of National Center for Biotechnology Information with BioProject ID PRJNA1072149 and PRJNA1032729.

## References

[B1] Babenko V. V. Podgorny O. V. Manuvera V. A. Kasianov A. S. Manolov A. I. Grafskaia E. N. (2020). Draft genome sequences of *Hirudo medicinalis* and salivary transcriptome of three closely related medicinal leeches. BMC Genomics 21, 331. 10.1186/s12864-020-6748-0 32349672 PMC7191736

[B2] Borda E. Siddall M. E. (2004). Arhynchobdellida (Annelida: Oligochaeta: Hirudinida): phylogenetic relationships and evolution. Mol. Phylogenetics Evol. 30, 213–225. 10.1016/j.ympev.2003.09.002 15022771

[B3] Castellano I. Merlino A. (2012). γ-Glutamyltranspeptidases: sequence, structure, biochemical properties, and biotechnological applications. Cell. Mol. Life Sci. 69, 3381–3394. 10.1007/s00018-012-0988-3 22527720 PMC11115026

[B4] Chen J. Xie X. Zhang H. Li G. Yin Y. Cao X. (2021). Pharmacological activities and mechanisms of hirudin and its derivatives - a review. Front. Pharmacol. 12, 660757. 10.3389/fphar.2021.660757 33935784 PMC8085555

[B5] Cheng B. Liu F. Gao T. Zhang K. Lu Y. (2025). A Whitmania pigra destabilase fusion protein and its application. China Patent No CN119080950A. Beijing: China National Intellectual Property Administration.

[B6] Cheng S. (2018). “Studies on polypeptide compositions and anticoagulant mechanism of leeches,” in Master degree. Wuhan, China: Hubei University of Chinese Medicine.

[B7] Chopin V. Salzet M. Baert J. Vandenbulcke F. Sautiére P. E. Kerckaert J. P. (2000). Therostasin, a novel clotting factor Xa inhibitor from the rhynchobdellid leech, *Theromyzon tessulatum* . J. Biol. Chem. 275, 32701–32707. 10.1074/jbc.M909217199 10852926

[B8] Commission C. P. (2020). Pharmacopoeia of the people’s Republic of China. Beijing: Medicine Science and Technology Press.

[B9] Ding Y. Duan T. Shan Y. Wang X. Yang H. Yuan R. (2016). Comparative studies on anti-thrombin activity and anticoagulant mechanism between *Whitmania pigra* Whitman and *Hirudinaria manillensis* . China Pharm. 19, 1621–1624.

[B10] Dunwiddie C. Thornberry N. A. Bull H. G. Sardana M. Friedman P. A. Jacobs J. W. (1989). Antistasin, a leech-derived inhibitor of factor Xa. J. Biol. Chem. 264, 16694–16699. 10.1016/s0021-9258(19)84761-0 2777803

[B11] Emms D. M. Kelly S. (2019). OrthoFinder: phylogenetic orthology inference for comparative genomics. Genome Biol. 20, 238. 10.1186/s13059-019-1832-y 31727128 PMC6857279

[B12] Flynn J. M. Hubley R. Goubert C. Rosen J. Clark A. G. Feschotte C. (2020). RepeatModeler2 for automated genomic discovery of transposable element families. Proc. Natl. Acad. Sci. U. S. A. 117, 9451–9457. 10.1073/pnas.1921046117 32300014 PMC7196820

[B13] Gao J. W. Sun J. W. Tong X. R. Wang H. Hu Q. M. Cao Y. R. (2023). Chromosome-level *Dinobdella ferox* genome provided a molecular model for its specific parasitism. Parasites and Vectors 16, 322. 10.1186/s13071-023-05837-7 37697397 PMC10494388

[B14] Guan D. L. Yang J. Liu Y. K. Li Y. Mi D. Ma L. B. (2019). Draft genome of the Asian buffalo leech *Hirudinaria manillensis* . Front. Genet. 10, 1321. 10.3389/fgene.2019.01321 32010187 PMC6977106

[B15] Guan S. Yuan Z. Zhou Y. Zhang Y. Ye X. Hu B. (2012). Comparative studies on anti-thrombus and anti-coagulation effects of *Hirudo* of different species. Chin. J. Hosp. Pharm. 32, 1093–1096. 10.13286/j.cnki.chinhosppharmacyj.2012.14.005

[B16] Gupta V. Sengupta M. Prakash J. Tripathy B. C. (2016). Production of recombinant pharmaceutical proteins. Basic Appl. Aspects Biotechnol. 1, 77–101. 10.1007/978-981-10-0875-7_4

[B17] He C. Chen X. Zhang X. Wang L. Hu S. (2021). A textual research on *Whitmania pigra* Whitman as the origin of *Hirudo* . China Med. Her. 18, 112–115. 10.20047/j.issn1673-7210.2021.24.028

[B18] He W. Yang J. Jing Y. Xu L. Yu K. Fang X. (2023). NGenomeSyn: an easy-to-use and flexible tool for publication-ready visualization of syntenic relationships across multiple genomes. Bioinformatics 39, btad121. 10.1093/bioinformatics/btad121 36883694 PMC10027429

[B19] Hong S. J. Kang K. W. (1999). Purification of granulin-like polypeptide from the blood-sucking leech, *Hirudo nipponia* . Protein Expr. Purif. 16, 340–346. 10.1006/prep.1999.1077 10419830

[B20] Hu J. Fan J. Sun Z. Liu S. (2020). NextPolish: a fast and efficient genome polishing tool for long-read assembly. Bioinformatics 36, 2253–2255. 10.1093/bioinformatics/btz891 31778144

[B21] Hu J. Wang Z. Sun Z. Hu B. Ayoola A. O. Liang F. (2024). NextDenovo: an efficient error correction and accurate assembly tool for noisy long reads. Genome Biol. 25, 107. 10.1186/s13059-024-03252-4 38671502 PMC11046930

[B22] Jiang Q. Wang L. Liu Q. Hu J. Li J. Zhang Y. (2020). Purification and characterization of a novel fibrinolytic enzyme from Whitmania pigra Whitman. Protein Expr. Purif. 174, 105680. 10.1016/j.pep.2020.105680 32497576

[B23] Jin J. J. Yu W. B. Yang J. B. Song Y. Depamphilis C. W. Yi T. S. (2020). GetOrganelle: a fast and versatile toolkit for accurate *de novo* assembly of organelle genomes. Genome Biol. 21, 241. 10.1186/s13059-020-02154-5 32912315 PMC7488116

[B24] Jung H. I. Kim S. I. Ha K. S. Joe C. O. Kang K. W. (1995). Isolation and characterization of guamerin, a new human leukocyte elastase inhibitor from Hirudo nipponia. J. Biol. Chem. 270, 13879–13884. 10.1074/jbc.270.23.13879 7775446

[B25] Kim Y. H. Choi J. G. Lee G. M. Kang K. W. (2001). Domain and genomic sequence analysis of bdellin-KL, a leech-derived trypsin-plasmin inhibitor. J. Biochem. 130, 431–438. 10.1093/oxfordjournals.jbchem.a003003 11530020

[B26] Krezel A. M. Wagner G. Seymour-Ulmer J. Lazarus R. A. (1994). Structure of the RGD protein decorsin: conserved motif and distinct function in leech proteins that affect blood clotting. Science 264, 1944–1947. 10.1126/science.8009227 8009227

[B27] Kvist S. Manzano-Marín A. De Carle D. Trontelj P. Siddall M. E. (2020). Draft genome of the European medicinal leech *Hirudo medicinalis* (Annelida, Clitellata, Hirudiniformes) with emphasis on anticoagulants. Sci. Rep. 10, 9885. 10.1038/s41598-020-66749-5 32555498 PMC7303139

[B28] Li D. Huang L. Qu X. (2013). Medicinal Fauna of China. Fuzhou: Fujian Science and Technology Press.

[B29] Li J. Yu X. Zhang J. Zhang T. Gong Y. Jin G. (2014). Anti-thrombin activity analysis and effects of cryopreservation on anticoagulant activity of four species of leeches. Fish. Sci. 33, 591–593. 10.16378/j.cnki.1003-1111.2014.09.012

[B30] Li W. Liao F. Yin X. Peng J. Ou X. Zhang Q. (1997). Experimental study on anti-platelet aggregation and anti-coagulation of seven spices of leech. Pharmacol. Clin. Chin. Materia Medica 13, 32–34.

[B31] Liu F. Yang D. (2014). Study on artificial breeding model of medical leech in China. World Sci. Technol. Mod. Traditional Chin. Med. Materia Medica 10, 2170–2173. 10.11842/wst.2014.10.020

[B32] Liu Z. Zhao F. Huang Z. He B. Liu K. Shi F. (2024). A chromosome-level genome assembly of the non-hematophagous leech *Whitmania pigra* (Whitman 1884): identification and expression analysis of antithrombotic genes. Genes 15, 164. 10.3390/genes15020164 38397154 PMC10887747

[B33] Liu Z. Zhao F. Huang Z. Hu Q. Meng R. Lin Y. (2023). Revisiting the Asian buffalo leech (*Hirudinaria manillensis*) genome: focus on antithrombotic genes and their corresponding proteins. Genes 14, 2068. 10.3390/genes14112068 38003011 PMC10671345

[B34] Marin E. Kornilov D. A. Bukhdruker S. S. Aleksenko V. A. Manuvera V. A. Zinovev E. V. (2023). Structural insights into thrombolytic activity of destabilase from medicinal leech. Sci. Rep. 13, 6641. 10.1038/s41598-023-32459-x 37095116 PMC10126035

[B35] Markwardt F. (2002). Hirudin as alternative anticoagulant - a historical review. Seminars Thrombosis Hemostasis 28, 405–414. 10.1055/s-2002-35292 12420235

[B36] Mcphalen C. A. Schnebli H. P. James M. N. (1985). Crystal and molecular structure of the inhibitor eglin from leeches in complex with subtilisin Carlsberg. FEBS Lett. 188, 55–58. 10.1016/0014-5793(85)80873-5 3926539

[B37] Michalsen A. Roth M. Dobos G. (2007). Medicinal leech therapy. Stuttgart: Georg Thieme Verlag.

[B38] Mittl P. R. Di Marco S. Fendrich G. Pohlig G. Heim J. Sommerhoff C. (1997). A new structural class of serine protease inhibitors revealed by the structure of the hirustasin-kallikrein complex. Structure 5, 253–264. 10.1016/s0969-2126(97)00183-4 9032072

[B39] Moser M. Auerswald E. Mentele R. Eckerskorn C. Fritz H. Fink E. (1998). Bdellastasin, a serine protease inhibitor of the antistasin family from the medical leech (*Hirudo medicinalis*) - primary structure, expression in yeast, and characterisation of native and recombinant inhibitor. Eur. J. Biochem. 253, 212–220. 10.1046/j.1432-1327.1998.2530212.x 9578479

[B40] Müller C. Wang Z. Hamann M. Sponholz D. Hildebrandt J. P. (2022). Life without blood: molecular and functional analysis of hirudins and hirudin-like factors of the Asian non-hematophagous leech *Whitmania pigra* . J. Thrombosis Haemostasis 20, 1808–1817. 10.1111/jth.15762 35587545

[B41] Nguyen L. T. Schmidt H. A. Von Haeseler A. Minh B. Q. (2015). IQ-TREE: a fast and effective stochastic algorithm for estimating maximum-likelihood phylogenies. Mol. Biol. Evol. 32, 268–274. 10.1093/molbev/msu300 25371430 PMC4271533

[B42] Ou X. Zhang H. Ding J. Liu Z. Zhang L. Yang T. (1996). Studies of the anticoagulant activity of 4 kind of leeches. Nat. Prod. Res. Dev. 2, 54–56. 10.16333/j.1001-6880.1996.02.012

[B43] Patro R. Duggal G. Love M. I. Irizarry R. A. Kingsford C. (2017). Salmon provides fast and bias-aware quantification of transcript expression. Nat. Methods 14, 417–419. 10.1038/nmeth.4197 28263959 PMC5600148

[B44] Perzborn E. Roehrig S. Straub A. Kubitza D. Misselwitz F. (2011). The discovery and development of rivaroxaban, an oral, direct factor Xa inhibitor. Nat. Rev. Drug Discov. 10, 61–75. 10.1038/nrd3185 21164526

[B45] Phillips A. J. Siddall M. E. (2009). Poly-paraphyly of Hirudinidae: many lineages of medicinal leeches. BMC Evol. Biol. 9, 246. 10.1186/1471-2148-9-246 19811660 PMC2768714

[B46] Qiao N. Bai Y. Wang G. Xu S. (2013). Comparative morphology study on the jaws of three leech species of the genus *Whitmania* Blanchard. Sichuan J. Zoology 32, 526–529. 10.3969/j.issn.1000-7083.2013.04.009

[B47] Ranwez V. Harispe S. Delsuc F. Douzery E. J. (2011). MACSE: multiple Alignment of Coding SEquences accounting for frameshifts and stop codons. PLoS One 6, e22594. 10.1371/journal.pone.0022594 21949676 PMC3174933

[B48] Rhie A. Walenz B. P. Koren S. Phillippy A. M. (2020). Merqury: reference-free quality, completeness, and phasing assessment for genome assemblies. Genome Biol. 21, 245. 10.1186/s13059-020-02134-9 32928274 PMC7488777

[B49] Sawyer R. T. (1986). Leech biology and behaviour. Oxford: Oxford University Press.

[B50] Seppey M. Manni M. Zdobnov E. M. (2019). BUSCO: assessing genome assembly and annotation completeness. Methods Mol. Biol. 1962, 227–245. 10.1007/978-1-4939-9173-0_14 31020564

[B51] Shen W. Le S. Li Y. Hu F. (2016). SeqKit: a cross-platform and ultrafast toolkit for FASTA/Q file manipulation. PLoS One 11, e0163962. 10.1371/journal.pone.0163962 27706213 PMC5051824

[B52] Sig A. K. Guney M. Uskudar Guclu A. Ozmen E. (2017). Medicinal leech therapy - an overall perspective. Integr. Med. Res. 6, 337–343. 10.1016/j.imr.2017.08.001 29296560 PMC5741396

[B53] Sommerhoff C. P. Söllner C. Mentele R. Piechottka G. P. Auerswald E. A. Fritz H. (1994). A Kazal-type inhibitor of human mast cell tryptase: isolation from the medical leech Hirudo medicinalis, characterization, and sequence analysis. Biol. Chem. Hoppe-Seyler 375, 685–694. 10.1515/bchm3.1994.375.10.685 7888081

[B54] Song J. Zhou Q. (1997). Discussion on the sources of shuizhi (leeches) in Chinese Pharmacopoeia. Lishizhen Med. Materia Medica Res. 8, 104.

[B55] Tang X. Chen M. Duan Z. Mwangi J. Li P. Lai R. (2018). Isolation and characterization of poecistasin, an anti-thrombotic antistasin-type serine protease inhibitor from leech *Poecilobdella manillensis* . Toxins 10, 429. 10.3390/toxins10110429 30373118 PMC6265900

[B56] Tong L. Dai S. X. Kong D. J. Yang P. P. Tong X. Tong X. R. (2022). The genome of medicinal leech (*Whitmania pigra*) and comparative genomic study for exploration of bioactive ingredients. BMC Genomics 23, 76. 10.1186/s12864-022-08290-5 35073842 PMC8787918

[B57] Wang X. Gan Q. Hu H. Hao J. Wu G. Gao Q. (2019). Measurement of antiplatelet aggregation and potency of *Hirudo* . Acta Pharm. Sin. 54, 2178–2183. 10.16438/j.0513-4870.2019-0147

[B58] Wang Y. Wang S. Liu Y. Xie Y. (2013). Effects of different processing methods on amino acid contents and anti-thrombin activity in leech. J. Traditional Chin. Med. Univ. Hunan 33, 42–45. 10.3969/j.issn.1674-070X.2013.11.012.042.04

[B59] Warkentin T. E. (2004). Bivalent direct thrombin inhibitors: hirudin and bivalirudin. Best. Pract. Res. Clin. Haematol. 17, 105–125. 10.1016/j.beha.2004.02.002 15171961

[B60] Yang T. (1996). Fauna sinica (Annelida Hirudinea). Beijing: Science Press.

[B61] Yu M. Su S. Zhou M. Cao M. Feng X. (2022). Identification of suspected species of *Hirudo nipponia* based on COI gene sequence. J. Southwest Univ. Nat. Sci. Ed. 44, 74–80. 10.13718/j.cnki.xdzk.2022.10.009

[B62] Zhang B. Wang B. Gong Y. Yu X. Lv J. (2012). Anticoagulant active substances extraction and anti-thrombin activity analysis of several species of leeches. Acta Sci. Nat. Univ. Sunyatseni 51, 92–96.

[B63] Zhang X. Zhuang Y. Li Z. Wu J. Zou J. Gao P. (2020). Comparative study on enzymatic hydrolysis processes of different radicals based on anticoagulant activity. Contemp. Chem. Ind. 49, 549–554. 10.13840/j.cnki.cn21-1457/tq.2020.03.011

[B64] Zhao F. Huang Z. He B. Liu K. Li J. Liu Z. (2024). Comparative genomics of two Asian medicinal leeches *Hirudo nipponia* and *Hirudo tianjinensis*: with emphasis on antithrombotic genes and their corresponding proteins. Int. J. Biol. Macromol. 270, 132278. 10.1016/j.ijbiomac.2024.132278 38750856

[B65] Zhao F. Jiang K. Su T. He B. Wu Q. Lin G. (2021). Phylogenomics of common species in Hirudiniformes. J. Jinggangshan Univ. Nat. Sci. 42, 41–47. 10.3669/j.issn.1674-8085.2021.04.009

[B66] Zheng J. Wang X. Feng T. Rehman S. U. Yan X. Shan H. (2022). Molecular mechanisms underlying hematophagia revealed by comparative analyses of leech genomes. Gigascience 12, giad023. 10.1093/gigascience/giad023 37039117 PMC10087013

[B67] Zhong M. Lei Y. Li Y. Tan H. Yuan R. (2020). *In vivo* anticoagulant activity of different concoctions of *Whitmania pigra* and *Hirudinaria manillensis* . J. Chin. Med. Mater. 43, 1351–1353. 10.13863/j.issn1001-4454.2020.06.012

[B68] Zhou C. Mccarthy S. A. Durbin R. (2023). YaHS: yet another Hi-C scaffolding tool. Bioinformatics 39, btac808. 10.1093/bioinformatics/btac808 36525368 PMC9848053

