# Supplementary material for: Comparative genomics of three non-hematophagous leeches (Whitmania spp.) with emphasis on antithrombotic biomolecules
Source: Front Genet. 2025 Feb 26;16:1548006. doi: 10.3389/fgene.2025.1548006 (PMC11897028; doi:10.3389/fgene.2025.1548006)
Supplement: Supplementary file 1 [file DataSheet1.zip › 00Supplementary_Figures_S1-S17.doc]

**Supplementary figures**

Note: for each figure, the first protein sequence was the previously identified archetypal protein; the red triangles show conserved cysteines; the black stars mean stop codons; the red circle(s) indicate the reactive or binding residue(s) identified in the archetypal protein. For details, see our previous paper: Liu Z, Zhao F, Huang Z, He B, Liu K, Shi F, Zhao Z, Lin G. A chromosome-level genome assembly of the non-hematophagous leech *Whitmania pigra* (Whitman 1884): identification and expression analysis of antithrombotic genes. Genes, 2024, 15: 164.

**Figure S1** Alignment of archetypal hirudin and hirudins from *Whitmania pigra*.

**Figure S2** Alignment of archetypal granulin and progranulins identified from *Whitmania* species.

**Figure S3** Alignment of antistasins.

**Figure S4** Alignment of lefaxins.

**Figure S5** Alignment of therostasins.

**Figure S6** Alignment of proteins from hirustasin superfamily (including hirustasin, bdellastasin, guamerin, piguamerin, and hirustasin-like families).

**Figure S7** Alignment of eglins.

**Figure S8** Alignment of bdellins.

**Figure S9** Alignment of LDTIs.

**Figure S10** Alignment of HMEIs.

**Figure S11** Alignment of saratins.

**Figure S12** Alignment of apyrases.

**Figure S13** Alignment of lumbrokinases.

**Figure S14** Alignment of destabilases.

**Figure S15** Alignment of GGTs.

**Figure S16** Alignment of LCIs.

**Figure S17** Alignment of hyaluronidases.
